# Distribution of Dengue Virus Types 1 and 4 in Blood Components from Infected Blood Donors from Puerto Rico

**DOI:** 10.1371/journal.pntd.0004445

**Published:** 2016-02-12

**Authors:** Germán Añez, Daniel A. R. Heisey, Caren Chancey, Rafaelle C. G. Fares, Luz M. Espina, Kátia P. R. Souza, Andréa Teixeira-Carvalho, David E. Krysztof, Gregory A. Foster, Susan L. Stramer, Maria Rios

**Affiliations:** 1 U.S. Food and Drug Administration, Silver Spring, Maryland, United States of America; 2 American Red Cross, Gaithersburg, Maryland, United States of America; Duke-NUS GMS, SINGAPORE

## Abstract

**Background:**

Dengue is a mosquito-borne viral disease caused by the four dengue viruses (DENV-1 to 4) that can also be transmitted by blood transfusion and organ transplantation. The distribution of DENV in the components of blood from infected donors is poorly understood.

**Methods:**

We used an in-house TaqMan qRT-PCR assay to test residual samples of plasma, cellular components of whole blood (CCWB), serum and clot specimens from the same collection from blood donors who were DENV-RNA-reactive in a parallel blood safety study. To assess whether DENV RNA detected by TaqMan was associated with infectious virus, DENV infectivity in available samples was determined by culture in mosquito cells.

**Results:**

DENV RNA was detected by TaqMan in all tested blood components, albeit more consistently in the cellular components; 78.8% of CCWB, 73.3% of clots, 86.7% of sera and 41.8% of plasma samples. DENV-1 was detected in 48 plasma and 97 CCWB samples while DENV-4 was detected in 21 plasma and 31 CCWB samples. In mosquito cell cultures, 29/111 (26.1%) plasma and 32/97 (32.7%) CCWB samples were infectious. A subset of samples from 29 donors was separately analyzed to compare DENV viral loads in the available blood components. DENV viral loads did not differ significantly between components and ranged from 3–8 log_10_ PCR-detectable units/ml.

**Conclusions:**

DENV was present in all tested components from most donors, and viral RNA was not preferentially distributed in any of the tested components. Infectious DENV was also present in similar proportions in cultured plasma, clot and CCWB samples, indicating that these components may serve as a resource when sample sizes are limited. However, these results suggest that the sensitivity of the nucleic acid tests (NAT) for these viruses would not be improved by testing whole blood or components other than plasma.

## Author Summary

Dengue is a febrile disease caused by the four dengue viruses (DENV-1 to 4) transmitted by mosquitoes from the genus *Aedes* that can also be transmitted by blood transfusion and organ transplantation. DENV is present in the blood of infected individuals without symptoms, meaning that infected donors may pose a risk to the safety of the donor blood supply. Current methods for detecting transfusion-transmitted viruses by nucleic acid testing use plasma as the testing specimen, and the number of tests that can be performed without reducing availability of blood for transfusion is limited. To determine whether blood components other than plasma could be suitable for testing, we quantified and compared the concentrations of DENV RNA in the residual components of blood collected from subjects previously identified as infected in a parallel blood safety study. Additionally, when available, samples were also evaluated for infectivity in tissue culture. The results showed that DENV RNA and infectious virions were detected comparably in all blood components, suggesting that using alternate specimens may improve sample availability but may not improve testing sensitivity.

## Introduction

Dengue is a febrile disease of global public health importance, affecting more than 100 tropical and subtropical countries and causing an estimated 390 million infections per year [1, 2]. The disease is caused by any of four closely related dengue viruses (DENV-1 to 4) from the genus *Flavivirus*, family *Flaviviridae*, and is transmitted by mosquitoes from the genus *Aedes*. Infection with any of the four DENV can be asymptomatic or cause an influenza-like illness (dengue fever), that may progress to a potentially life-threatening condition (severe dengue or dengue hemorrhagic fever; DHF), especially after secondary, heterotypic infections [3, 4]. Although dengue primarily affects tropical and sub-tropical countries, the virus can be imported by infected travelers to non-endemic regions. Recent dengue epidemics have occurred in non-endemic areas of Europe and the United States of America (U.S.), where the transmitting mosquitoes have become established, enabling local transmission cycles [5–7]. Dengue is endemic in the U.S. territory of Puerto Rico, where it has caused large epidemics in 2010 (>26,700 suspected cases), 2012 (>12,800 cases), 2013 (>18,100 cases) and 2014 (>8,600 cases), for a total of more than 66,000 suspected cases reported during these last four epidemic years [5, 8].

Transfusion-transmitted DENV (TT-DENV) has been reported in dengue endemic regions including Puerto Rico, where a transfusion of red blood cells caused DHF in a recipient [9–16]. DENV is also transmissible by solid organ transplant, thus posing a risk for recipients of these products [17–20]. At this time, there is no Food and Drug Administration (FDA)-approved nucleic acid test (NAT) for the screening of blood for DENV RNA. DENV, like many other arboviruses, can cause asymptomatic infections in up to 80% of infected individuals [21]. Prevalence studies conducted in endemic regions have found rates of asymptomatic infection among blood donors ranging from 0.07–0.45% [11, 12, 14, 16, 22, 23]. In 2010, Puerto Rico experienced a large epidemic with over 26,000 reported dengue cases [5, 24] and blood donations were screened for DENV using an antigen-based immunoassay (DENV NS1 Ag) under an FDA-approved investigational new drug (IND) protocol [5, 25]. Using this protocol, DENV was detected in blood units collected from asymptomatic blood donors from Puerto Rico and from Key West, Florida. Data acquired during that IND study indicated that the DENV NS1 Ag assay lacked proper sensitivity and specificity for blood donor screening [16].

Antibody tests for DENV are available and used for diagnostic purposes, but they are unsuitable for blood screening because the infectious viremic stage precedes seroconversion. Moreover, unlike chronic infections, identification of DENV-specific antibodies does not necessarily represent active viral infection. In addition, infection and antibody development can occur in the absence of clinically apparent infection. Therefore, NAT targeting DENV RNA has been considered the most appropriate approach for blood screening. Although there is no FDA-approved assay for the screening of blood for DENV, during the epidemic seasons of 2012–2013 NAT using transcription-mediated amplification (TMA) was used to test blood donation samples in Puerto Rico under an IND protocol [16, 26].

In the absence of licensed DENV NAT for blood screening, developing measures to mitigate risk of TT-DENV and safeguard the blood supply is a challenge; even more so in endemic areas like Puerto Rico, and in areas with recurrent small outbreaks like Florida and Texas. The measures to be taken rely heavily on published data regarding the biology of infection, which are mostly obtained from clinical cases from epidemic areas. While the clinical course of dengue disease has been well-studied, many aspects of DENV infection in asymptomatic or pre-symptomatic infections are unknown or poorly understood. In particular, understanding the distribution of virions in the components of whole blood in asymptomatic or pre-symptomatic infections is critical to establishing and optimizing blood testing protocols, thus enabling the use of currently-discarded components to enhance sample availability.

In this study, we used residual samples from specimens from blood donors confirmed positive for DENV RNA that were initially reactive by TMA performed as part of an IND study in Puerto Rico [16, 26], to determine the distribution of DENV in blood components, quantify viral loads during the asymptomatic period, and demonstrate infectivity of the various components *in vitro*. In addition, we investigated whether, as has been observed for WNV [27–29], testing in blood components other than plasma or serum would result in increased assay sensitivity and enhancement of DENV detection.

## Materials and Methods

### Ethics statement

The protocol for specimen collection and testing by investigational and research assays was approved by the American Red Cross (ARC) Institutional Review Board. The study protocol (13-001B) was reviewed and considered exempt by the Research Including Human Subjects Committee at the U.S. FDA.

### Study population

This study was performed using available residual samples from blood donors who tested initially reactive for DENV RNA by an investigational NAT assay (DENV TMA assay, Hologic, Inc., formerly Gen-Probe, Inc.), in use under an FDA-approved IND to screen blood donations from Puerto Rico for DENV RNA as part of an independent blood safety study [16, 26]. Date of specimen collection and demographical information were available for all blood donors. All subjects were asymptomatic at the time of donation. Paired EDTA whole blood samples and their corresponding plasma samples were received for a total of 165 donors. Additionally, 149 clot and 155 serum specimens from those donors were available for testing. Samples were collected between August 2012 and August 2013, and the residual samples from tubes used for blood screening purposes by the ARC were unlinked and shipped either refrigerated (for collection tubes containing EDTA whole blood or blood clots) or frozen (for tubes containing plasma or serum) to the laboratory at the FDA for processing and testing.

The cellular components of whole blood (CCWB) specimens consisted of all blood cells from the EDTA blood sample tube remaining after removal of the plasma for blood testing purposes including NAT. Plasma specimens were obtained from the co-component units from that same collection, which, after testing positive for DENV RNA, were discarded from the blood supply and labeled for research use only. Blood clots and serum specimens were available as residuals of samples tested for DENV antigen and serology as part of the IND protocol.

Upon receipt, frozen specimens (plasma and serum) were thawed and divided into aliquots. One aliquot was used for cultivation, and the remaining aliquots were stored frozen at -80°C until processing. CCWB samples were prepared by centrifugation at 4°C, 3000 rpm, for 10 minutes, followed by removal of any remaining plasma, and divided into aliquots of 250 μL; one aliquot was used for cultivation and the remaining aliquots were mixed with 1 mL of Trizol and frozen at –80°C until RNA extraction. The blood clots were stored at 4°C until processing for cultivation and RNA extraction as described below.

Negative control plasma and CCWB specimens were obtained from non-DENV-endemic areas of the contiguous U.S. and were processed and extracted as described above.

### RNA extraction and real-time polymerase-chain reaction (qRT-PCR)

For liquid specimens (i.e. plasma, serum and cell culture supernatants), total RNA was extracted from aliquots of 140 μL from each sample using the QIAamp Viral Mini Kit (Qiagen) and eluted in either 70 or 140 μL of AVE buffer. Eluted RNA was kept at –80°C until testing.

Total RNA extraction from CCWB and clot samples was performed using Trizol reagent (Invitrogen) as per the manufacturer’s instructions with some modifications. Briefly, the 250 μL CCWB aliquots previously mixed and stored frozen with Trizol were allowed to thaw at room temperature and the RNA extraction processed as per the manufacturer’s protocol, followed by RNA cleaning and concentration using the RNeasy Mini kit (Qiagen), elution in 50 μL of RNase-free water and storage at –80°C until testing.

Approximately 150 mg of each clot were placed into an RNAse-free microcentrifuge tube and 1.5 ml of Trizol were added and vortex-mixed. The mixture was transferred to a homogenization vial containing ceramic beads (Precellys) and was subjected to a homogenization cycle (2 cycles of 20 seconds at 5,000 rpm with a 5 second break between cycles) in the Precellys 24 Dual homogenizer (Precellys). The clot-Trizol homogenate was then transferred to an RNase-free microcentrifuge tube and the RNA extraction protocol with Trizol was continued as described above. RNA was eluted in 50 μL of RNase-free water and stored at –80°C until testing.

RNA extracts were tested by an in-house dengue specific real-time polymerase-chain reaction (TaqMan) assay based on a modification of the protocol published by Johnson et al. [30]. Briefly, 10 μL of eluted RNA from each tested component were added to a reaction containing 12.5 pmol of primers and 6.25 pmol of probe for each DENV type in singleplex format in duplicate in a volume of 25 μL, using the 1-step RNA-to-Ct kit (Applied Biosystems). Reactions were conducted in a 7300 Real-time PCR System (Applied Biosystems). Standard curves for each DENV type, calibrated by endpoint dilution and covering a dynamic range of 7-log_10_, were used and results were reported as PCR-detectable units per ml (PDU/mL) for RNA extracted from liquid or CCWB specimens or per mg (PDU/mg) for RNA extracted from clots [31, 32]. Correction for extraction and elution volumes was done by using the formula: PCR detectable units per reaction x (1 ml [or 1 mg for clots]/sample extracted volume) x (elution volume/tested volume). At least two independent RNA extractions and TaqMan tests were conducted for each blood component evaluated. Samples with cycle threshold (Ct)<38 were considered positive [30]. Although samples with cycle threshold (Ct) ≥38 were not used for quantitative analysis, they were considered DENV RNA-positive if they tested positive in the co-component or were infectious in cell culture.

### Cell culture

The different blood components (i.e. plasma, serum, CCWB and clot) were used to infect monolayers of *Aedes albopictus* C6/36 cells in culture. The following volumes from each sample type were used for culture: 250 μL of undiluted cell suspension for CCWB; 500 μL of 1:20 diluted plasma or serum in MEM containing 2% FBS and 1% of fungizone and gentamycin (MEM2%); 150 mg of clots homogenized in 500 μL of MEM2% by extrusion using a syringe and needle. Dilution of liquid samples was performed to prevent non-specific cytopathic effect in the C6/36 monolayers. Samples for culture were added to T25 flasks containing ~85% confluent cell monolayers and incubated for one hour at 32°C and 5% CO_2_ with gentle rocking every 15 minutes. After incubation, 5 mL of MEM2% were added to each flask and the cell cultures were incubated at 32°C, 5% CO_2_, and monitored daily. Cell cultures exposed to CCWB had their inocula removed and replaced with fresh MEM2% media 24 h post-infection and were maintained as indicated above. On day 7 post-infection (d.p.i.), all cell cultures had their supernatants harvested and subjected to RNA extraction for testing by TaqMan as described above. An aliquot of supernatant was also used for titration of infectivity by focus-forming assay (FFA) using anti-DENV specific monoclonal antibody 4G2 obtained from hybridoma culture supernatants (ATCC), essentially as described previously [33]. Infectivity titers were reported as focus-forming units/ml (FFU/ml).

### Statistical analysis

RNA testing results were log-transformed to ensure normal distribution of the data and expressed as mean ± standard deviation. Differences between viral load or infectivity titer averages in the tested components from each DENV type were determined by *t*-test or by one-way ANOVA with Bonferroni’s post-test using GraphPad Prism version 5.04 (GraphPad Software, San Diego, USA). Statistical significance was considered with *p*<0.05.

## Results

### DENV RNA is detected by TaqMan in both plasma and cellular components of whole blood

Plasma samples from 165 Puerto Rican blood donors that tested initially reactive for DENV RNA by TMA assay as part of a parallel IND blood safety study were tested by an in-house TaqMan RT-PCR assay. A total of 69 (41.8%) of these 165 samples were also DENV RNA-reactive on our assay. Of these 69 samples, 48 (69.6%) tested reactive to DENV-1 and 21 (30.4%) tested reactive for DENV-4. Among the 165 CCWB tested, 130 (78.8%) were DENV RNA-reactive on our assay; 98 (75.4%) were reactive to DENV-1, 31 (23.8%) for DENV-4, and one (0.8%) was found to be reactive to both DENV-1 and DENV-4, suggesting a co-infection (Table 1). All negative control plasma and CCWB specimens were non-reactive by TaqMan.

**Table 1:** Summary of DENV testing by TaqMan in plasma, cellular component of whole blood, serum and clot specimens from blood donors from Puerto Rico, 2012–2013, n = 165, and negative controls from the contiguous U.S., n = 16.

	Plasma	CCWB	Serum	Clots
**DENV-1**				
With Ct≤38	32/48	84/99^#^	15/16	41/50
	(66.7%)	(85%)	(93.8%)	(82%)
With Ct>38	16/48	15/99^#^	1/16	9/50
	(33.3%)	(15%)	(6.3%)	(18%)
**DENV-4**				
With Ct≤38	20/21	30/32^#^	9/10	16/16
	(95%)	(93.8%)	(90%)	(100%)
With Ct>38	1/21	2/32^#^	1/10	0/16
	(5%)	(6.3%)	(10%)	(0%)
**ALL DENV**				
With Ct≤38	52/69	113/130	24/26	57/66
	(75.4%)	(86.9%)	(92.3%)	(86.4%)
With Ct>38	17/69	17/130	2/26	9/66
	(24.6%)	(13.1%)	(7.7%)	(13.6%)
Total reactive	69/165	130^#^/165	26/30	66/90
	(41.8%)	(78.8%)	(86.7%)	(73.3%)
**CONTROLS**				
Negative controls	0/16	0/16	ND	ND
	(0%)	(0%)		

# includes one sample reactive to both DENV-1 and DENV-4 by TaqMan.

**Fig 1:**
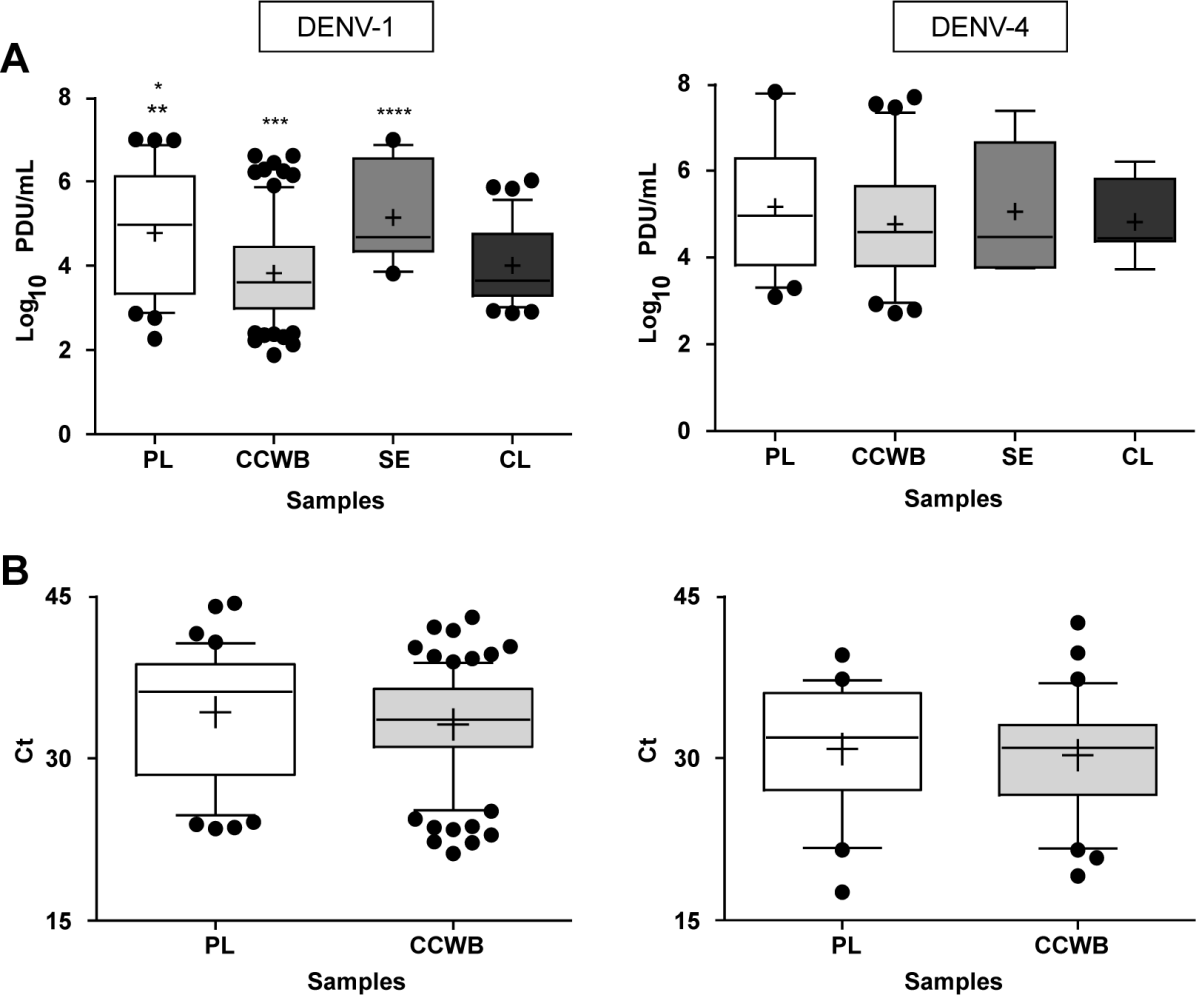
Detection of DENV RNA in blood components. A) DENV-1 and DENV-4 average viral load observed in plasma (PL), the cellular component of whole blood (CCWB), serum (SE) and homogenized clot specimens (CL). DENV viral load average in each sample co-component is expressed in log_10_ PDU/mL. B) Ct value averages for DENV-1 and DENV-4 plasma (PL) and cellular component of whole blood (CCWB). Samples were collected from infected Puerto Rican blood donors, 2012–2013. Only samples with Ct values ≤38 were included in the quantitative analysis. Whiskers represent 10-90^th^ percentile. Outliers are shown as black dots. Mean is shown as a “+” sign and median as a horizontal line inside the box. Statistical significance is indicated as follows: *p<0.001 vs CCWB; **p<0.05 vs CL; ***p<0.001vs SE; ****p<0.05 vs CL.

Quantitative analysis was performed for all DENV RNA-positive samples by TaqMan with Ct≤38 [30]. The cutoff of Ct≤38 was chosen because reactivity outside of the linear range of the standard curve produces unreliable detection, inappropriate for quantification. Most plasma and CCWB samples had Ct≤38; 75% and 87%, respectively (Table 1). The average Ct value for DENV-1-positive plasma samples (Ct≤38) was 31.3 ± 5.0 (range 23.5–38) for an average titer of 4.778 ± 1.416 (range 2.272–6.994) log_10_ PDU/mL, while the average Ct value for DENV-1-positive CCWB samples (Ct≤38) was 32 ± 4.2 (range 21.2–37.8) for an average titer of 3.824 ± 1.168 (range 1.884–6.610) log_10_ PDU/mL. For DENV-4-positive plasma samples (Ct≤38) the average Ct value was 30.4 ± 5.6 (range 17.6–37.3), with average titer of 5.163 ± 1.552 (range 3.097–8.324) log_10_ PDU/mL, while the DENV-4-positive CCWB samples (Ct≤38) had an average Ct value of 29.5 ± 4.6 (range 19.1–37.3) for an average titer of 4.761 ± 1.356 (range 2.716–7.691) log_10_ PDU/mL (Fig 1).

### DENV RNA is detected by TaqMan in blood clots and serum specimens

Laboratory diagnosis of DENV is mostly performed using serum samples, and the blood clot from which the serum is recovered is usually discarded. When available, we have also tested sera and their respective blood clots for determination of DENV viral loads and infectivity, with the aim of defining the specimen that produces the highest rate of detection for DENV RNA as the most suitable testing sample for both diagnostic and blood screening settings.

To assess DENV viral loads in clots, we standardized a process for extracting RNA from homogenized blood clots, and determined viral loads in clots from 90 samples that had tested positive for DENV RNA on plasma and/or CCWB by our TaqMan assay and had clots available for testing. Of these 90 clot homogenates, 66 (73.3%) were TaqMan-reactive, of which 50/66 (75.8%) were DENV-1 and 16 (24.2%) were DENV-4. Most tests (82%) produced reactivity with Ct≤38 (Table 1). A total of 30 serum samples from the same donors who had the clots tested were available for testing. Of those, 26 (86.7%) tested reactive to DENV, of which 16/26 (61.5%) were reactive to DENV-1, while 10/26 (38.5%) were reactive to DENV-4 (Table 1).

Overall, there was a correlation between the DENV viral loads found among the liquid components (serum and plasma) and among the cellular components (clots and CCWB). However, DENV-1 viral loads were significantly higher in the liquid components (plasma and serum) than those found in cellular components, CCWB (*p*≤0.001) and clots (*p*≤0.05); while DENV-4 viral loads did not differ significantly between samples from different blood components tested (Fig 1).

### DENV viral loads are equivalent among blood components tested when all component viral loads are quantifiable, even after long-term refrigerated storage of clots

A side-by-side comparison of quantifiable DENV viral loads in all blood components from a subset of DENV-infected blood donors was performed to investigate whether, as reported for WNV, DENV would have a higher viral load associated with the cellular components of the blood. As stated above, quantitative analysis was only performed when all available specimens from each donor produced quantifiable TaqMan results with Ct values of 38 or lower (Ct≤38), which falls within the linear range of the standard curve.

**Fig 2:**
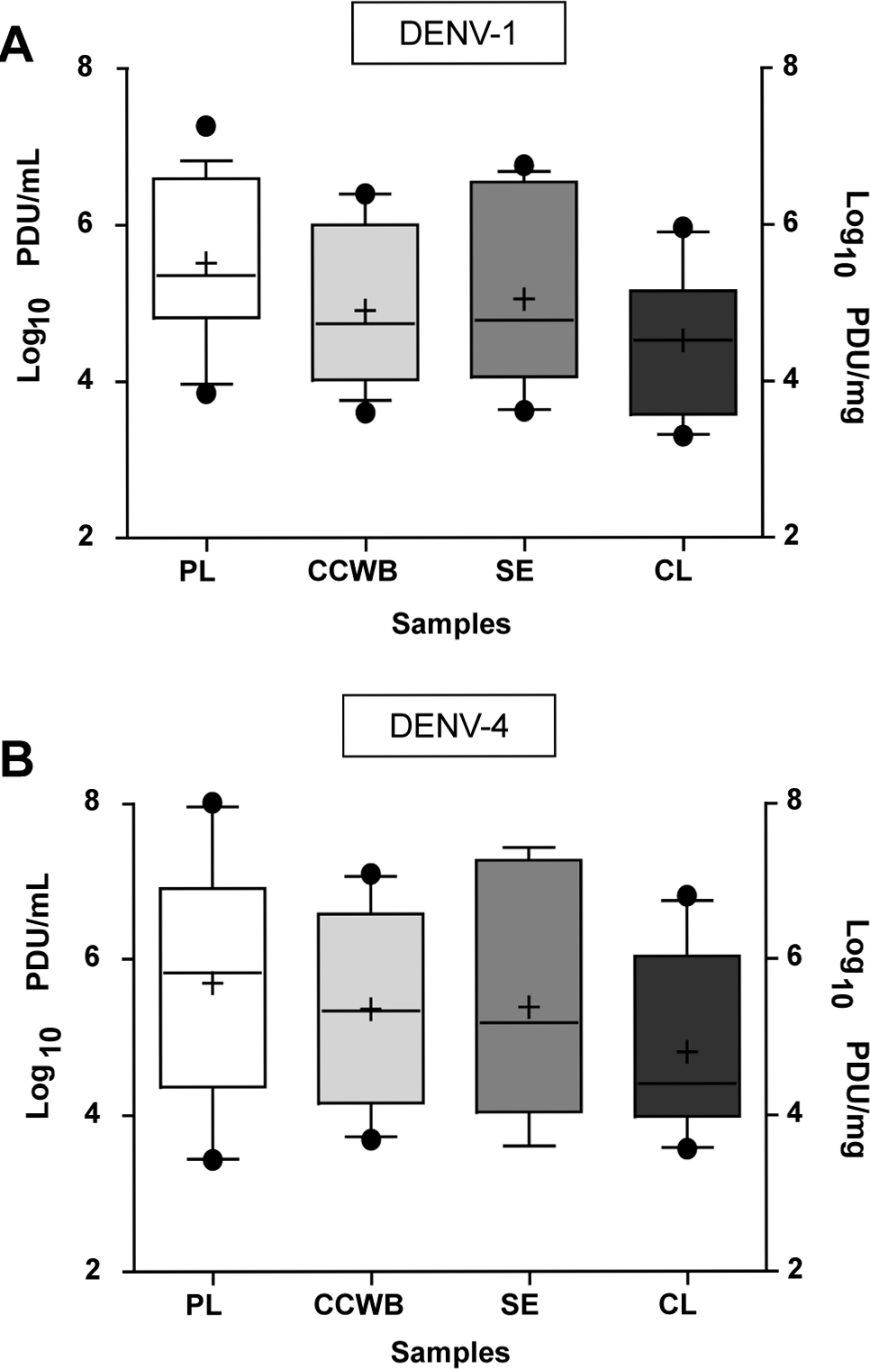
Mean DENV-1 and DENV-4 viral loads in blood components. A) DENV-1 and B) DENV-4 viral load averages observed in plasma (PL) (n = 29), the cellular component of whole blood (CCWB) (n = 29), serum (SE) (n = 21) and clots (CL) (n = 21) from samples from infected Puerto Rican blood donors, 2012–2013. Whiskers represent 10-90th percentile. Outliers are shown as black dots. Mean is shown as a “+” sign and median as a horizontal line inside the box.

A subset of 29 donors fulfilled this criterion for plasma and CCWB, of whom 19 (65.5%) had DENV-1 RNA and 10 (34.5%) had DENV-4 RNA detected in their components. DENV viral loads did not differ significantly among the tested components (ANOVA *p*>0.05), ranging from 3.6–6.4 (average ± SD: 5.0 ± 1.0) log_10_ PDU/mL in CCWB and 3.9–7.3 (average ± SD: 5.5 ± 1.0) log_10_ PDU/ml in plasma. Similar results were obtained for samples from donors infected with DENV-4; 3.7–7.1 (average ± SD: 5.4 ± 1.4) log_10_ PDU/mL in CCWB and 3.4–8.0 (average ± SD: 5.7 ± 1.5) log_10_ PDU/mL in plasma and. Results from the DENV RNA quantitation in this group are shown in Fig 2 and S1 Fig and S1 Table.

**Fig 3:**
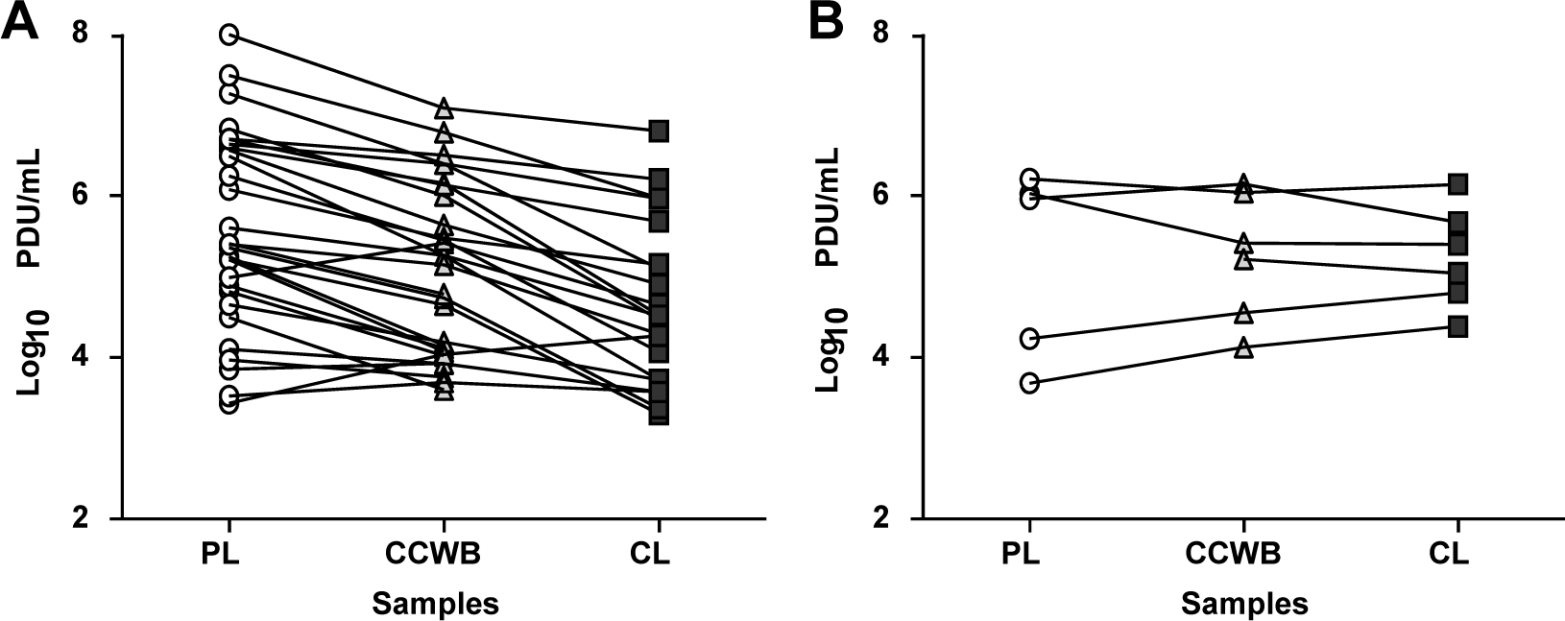
Linked comparison of DENV viral loads in blood components including clots. A) DENV-1 and DENV-4 combined viral loads in plasma (PL), the cellular component of whole blood (CCWB) and clot (CL), n = 29 samples. B) DENV-1 and DENV-4 combined viral loads in plasma (PL), the cellular component of whole blood (CCWB) and fresher clot (CL), n = 6 samples.

Of the 29 samples chosen for this analysis, 21 had serum and clot specimens available for testing. Serum viral loads did not differ significantly from plasma and CCWB viral loads, ranging from 3.6–6.8 (average ± SD: 4.9 ± 0.9) log_10_ PDU/mL for DENV-1 and 3.6–7.4 (average ± SD: 5.4 ± 1.1) log_10_ PDU/mL for DENV-4. Notably, the clots tested for this analysis had been stored at 4°C since collection (range 3.5–10 months of age). Surprisingly, all 21 tested clots contained detectable DENV RNA although at values slightly lower to those obtained from testing the other components. The viral loads detected from the clot homogenate extracts ranged from 3.3–6.0 (average ± SD: 4.5 ± 0.9) log_10_ PDU/ml for DENV-1 and of 3.5–6.8 (average ± SD: 4.8 ± 1.0) log_10_ PDU/ml for DENV-4 even after prolonged storage at 4°C, suggesting that only limited degradation of the viral RNA in the clot occurred during storage (S1 Table, Figs 2 and 3A).

The age of clot at the time of testing weakly correlated with the DENV viral loads detected, with fresher clots containing higher DENV viral loads. To confirm this apparent association, we tested an additional six fresher (≤2.5 months of storage at 4°C) clot specimens that were available. We found that the DENV viral loads in these six clots were comparable and in some cases higher than those obtained from the plasma or CCWB co-component; of these, four were reactive for DENV-1 and two for DENV-4. DENV-1 viral loads in these clots ranged from 4.3–5.7 (average ± SD: 4.9 ± 0.5) log_10_ PDU/mL, and for DENV-4 from 4.8–6.1 (average ± SD: 5.4 ± 0.7) log_10_ PDU/mL (S2 Table, Figs 3B and S2). The overall results indicate that a higher concentration of DENV RNA is present in fresher clots (average storage age DENV-1: 1.32 months, DENV-4: 0.77 months) in comparison to that observed in clots stored for longer periods of time (average storage age DENV-1: 7.39 months, DENV-4: 5.82 months). This observation allows us to speculate that clots may harbor a high concentration of virions attached to or inside cells and although the viral load tends to drop over time, DENV RNA can still be detectable after 3 to 10 months of storage at 4°C (S1 and S2 Tables).

### Infectious DENV is detected by mosquito cell co-culture in all blood components

**Fig 4:**
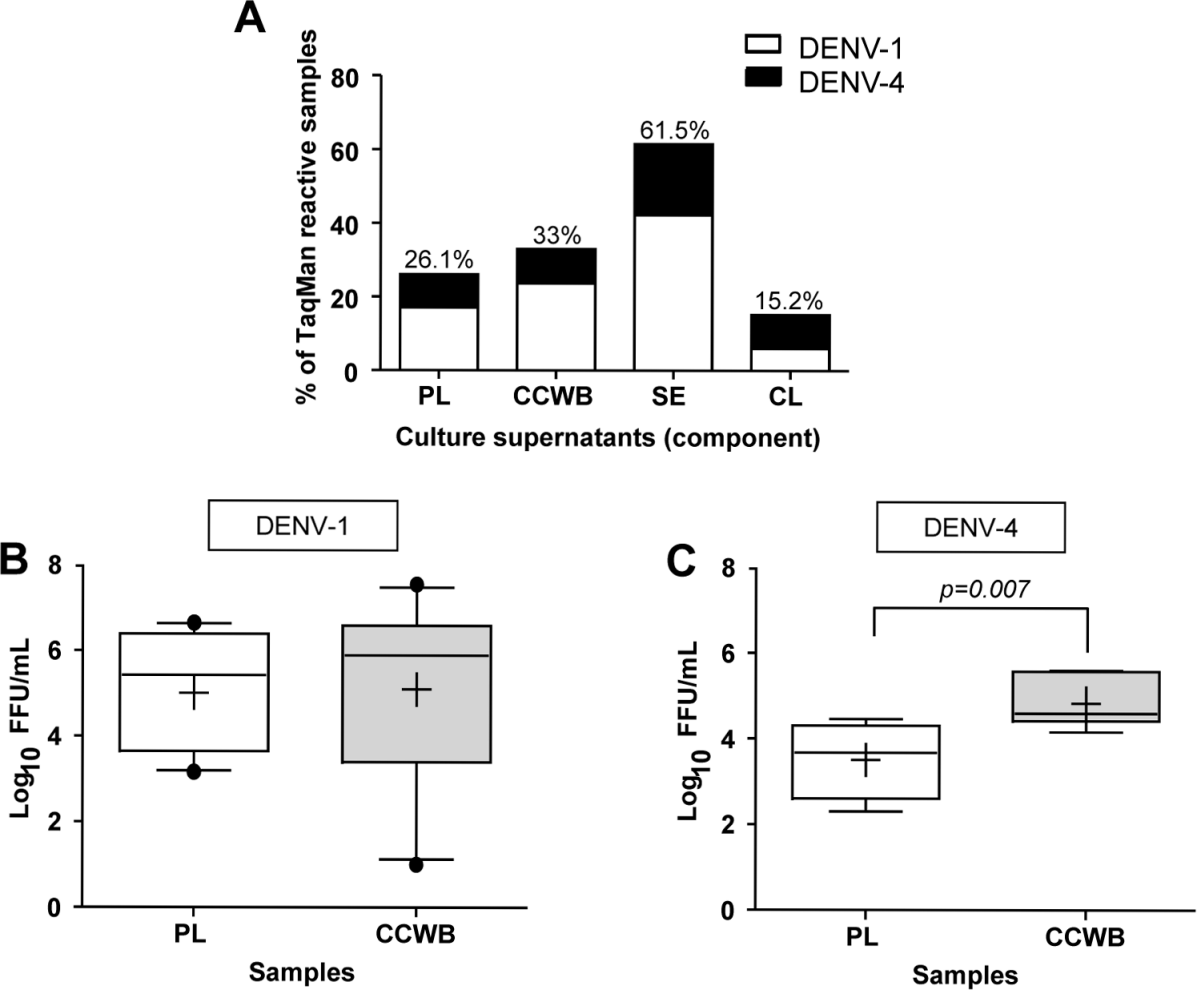
Culture of DENV-positive blood components. A) Percentage of TaqMan-reactive cell culture supernatant from mosquito C6/36 cells infected with plasma (PL), the cellular component of whole blood (CCWB), serum (SE) or homogenized clots (CL) from samples from Puerto Rican blood donors, 2012–2013. B) Infectivity titers of DENV-1-positive PL or CCWB from samples from infected Puerto Rican blood donors, 2012–2013, in supernatants from a single passage in cultured mosquito C6/36 cells. C) Infectivity titers of DENV-4-positive PL or CCWB from samples from infected Puerto Rican blood donors, 2012–2013, in supernatants from a single passage in cultured mosquito C6/36 cells. For both B) and C), whiskers represent 10-90th percentile. Outliers are shown as black dots. Mean is shown as a “+” sign and median as a horizontal line inside the box.

Plasma and CCWB samples were also cultured in the C6/36 mosquito cell line if sufficient volumes were available, in order to verify infectivity of DENV present in these components. Culture positivity was determined by TaqMan assay performed on cell culture supernatants. Of 111 plasma samples cultured, 29 supernatants (26.1%) tested TaqMan-reactive, 19 for DENV-1 and 10 for DENV-4; while 32/97 (33.0%) of CCWB culture supernatants tested TaqMan-reactive, 23 for DENV-1 and 9 for DENV-4 (Fig 4A).

Most specimens that were positive by tissue culture had also been reactive in the original tested component by TaqMan. For plasma specimens, 27/60 (45%) component TaqMan-reactive specimens were positive by tissue culture (18/44 DENV-1 and 9/16 DENV-4), and 2/51 (3.9%) cultures for which the component specimens were TaqMan-negative grew in culture (1 DENV-1 and 1 DENV-4). For CCWB specimens, 32/86 (37.6%) component TaqMan-reactive specimens were positive by tissue culture (23/65 DENV-1 and 9/22 DENV-4 including one specimen that was dual-positive by TaqMan but negative by culture), and 0/11 (0%) TaqMan-negative components produced infectious virus. In all cultures of TaqMan-reactive components, the DENV serotype, identified by TaqMan performed on the culture supernatant, matched that of the original component.

Owing to the low rates of recovery for TaqMan-negative plasma and CCWB, only TaqMan-reactive serum and clot specimens were tested in tissue culture. Of 26 serum supernatants cultured, 16 (61.5%) tested TaqMan-reactive, 11 DENV-1 and 5 DENV-4 (Fig 4A).

Clot homogenates that tested reactive to DENV RNA by TaqMan (n = 66) were also cultured to determine whether infectious particles persisted after long-term storage at 4°C. Ten (15.2%) clot homogenate culture supernatants tested TaqMan-reactive, 4 DENV-1 and 6 DENV-4 (Fig 4A). A subset of TaqMan-reactive supernatants from plasma and CCWB co-cultures were tested by FFA to confirm that cultures positive for DENV RNA by TaqMan also produced infectious virions. A total of 22 supernatants from cells cultured with TaqMan-reactive plasma samples (18 DENV-1 and 4 DENV-4) and 28 supernatants from cells cultured with TaqMan-reactive CCWB samples (22 DENV-1 and 6 DENV-4), yielded infectious virus as determined by the FFA assay (Fig 4B and 4C). FFA performed on culture supernatants of CCWB specimens from DENV-4-positive blood donors produced significantly more infectious virus than cultures of plasma specimens (mean 4.84 vs 3.51, p = 0.007), but there was no difference in the amount of infectious virus produced between CCWB and plasma from DENV-1-positive blood donors (Fig 4B and 4C).

## Discussion

Dengue is the most important arboviral disease in the world and has become one of the most frequently reported travel-associated infections worldwide [2, 4, 34]. DENV can be transmitted by blood transfusion and solid organ transplants, and all blood products used in clinical practice, namely packed red blood cells, platelet concentrates and fresh frozen plasma, have been implicated in the transfusion-transmission of DENV [9, 10, 13–20]. Because approximately 80% of DENV infections can be asymptomatic [21], DENV poses a threat for the blood supply, especially during outbreaks [11, 12, 14, 16, 22, 23, 35]. Overall, little is known about the biology of infection and pathogenesis of asymptomatic cases, especially regarding viral distribution in circulating blood, which is relevant for blood screening protocols.

DENV has been endemic in Puerto Rico for many years, and during the most recent epidemic years (2010, 2012, 2013 and 2014) more than 60,000 cases of dengue have been reported [5, 8, 24]. DENV-1 and DENV-4 were the viruses predominantly detected in Puerto Rico during the epidemic years 2012–2013 [8] and from samples collected in this study (Table 1).

Imported cases of dengue are reported every year in the U.S., and autochthonous dengue transmission has been documented in the states of Florida, Texas, Hawaii and New York [5, 36]. In the U.S., DENV has been demonstrated to circulate among asymptomatic blood donors in endemic (i.e., Puerto Rico) and non-endemic (i.e., Florida) areas experiencing outbreaks [14, 37]. Moreover, imported asymptomatic infections of travelers returning from endemic/outbreak areas may occur which are not identified and reported, and thus may serve as a source of localized outbreaks and pose an unknown level of risk to the blood supply. To better understand aspects of asymptomatic DENV infection which may affect the performance of blood screening assays, we analyzed the distribution of infectious DENV particles and viral RNA loads among different blood components (i.e., plasma, the cellular fraction of anticoagulated blood, serum and blood clots) in infected blood donors from Puerto Rico. The distribution of both DENV RNA and infectious particles and their associated viral loads in blood may provide information to help increase the sensitivity of viral detection and assess relative transfusion-transmission risks for each component of blood.

One of the main aims of this work was to determine the feasibility of use of samples other than plasma for the detection and quantification of DENV RNA. Probably due to easier collection logistics, serum has been the sample of choice for diagnosis of infection by DENV primarily in endemic areas where resources may be limited; however, plasma samples are also used although less frequently [4]. Conversely, FDA-approved blood donation screening assays for viruses such as West Nile Virus (WNV), Human Immunodeficiency Virus, Hepatitis C Virus (HCV) and Hepatitis B Virus, and the investigational DENV NAT assay are performed using plasma as the testing specimen, because plasma is easy to handle and store and readily available for testing consistent with other infectious disease markers [16]. Additionally, hemoglobin interferes with most NAT assays. However, there has been no direct comparison of efficiency of detection of DENV for various sample types in diagnostic or blood screening settings.

We compared the DENV RNA titers obtained from the plasma and serum with the cellular components of blood from all available DENV-TMA-reactive samples obtained from the ARC and found that at least for DENV-1 there is a significantly higher DENV viral load in the liquid components (plasma and serum) compared to those in the cellular components (CCWB and clots) (Fig 1). These differences between components observed for DENV-1 but not for DENV-4, may correspond to differences in the performance of our TaqMan assay for each virus, to an intrinsic difference in the natural distribution of these two viruses in blood components, or to non-viral factors such as prior donor exposure to DENV or timing of donations. When a more detailed quantitative analysis of DENV RNA was performed in a subset of plasma and CCWB samples with Ct≤38 as the cutoff in our TaqMan assay, those differences were not detected and similar DENV viral loads were observed across all tested infected individuals (Fig 2). Our results suggest that DENV RNA may be equally distributed in all blood components, thus reinforcing the notion that blood products from viremic donors may be equally infectious for recipients, despite the presence of anti-DENV antibodies, as >90% of infected blood donors in Puerto Rico are antibody-positive [35].

The flaviviruses DENV and WNV have both been linked to cases of transmission by tissue and organ transplant [17–20, 38, 39]. We attempted to use blood clots (acquired from tubes of blood collected without anticoagulants to obtain serum for screening purposes and other testing), as a source for the detection of DENV RNA and for its use for isolation of infectious viruses. DENV viral loads were in general lower in clots than in the other tested components, although not significantly (Fig 2). A surprising finding was the fact that we were able to detect DENV RNA and isolate viable viruses from clots that have been stored for several months at 4°C, thus suggesting a protective factor within the clot maintaining the integrity of virions and the retention of infectivity. This observation has prompted us to propose the use of fresh blood clots as a surrogate for tissues for the study of viral distribution in tissues and organs in research studies analyzing the infectivity of DENV and other viruses in tissues intended for transplantation.

Although we tried to culture the CCWB and clots as soon as they were received, in some circumstances some samples were stored for longer periods at 4°C than others before culture, and therefore it is hard to make an accurate side-by-side comparison between the efficiency of these components for DENV isolation, with respect to the liquid co-components (plasma and serum) that were received and kept frozen until infection. Still, our observation of substantial culture positivity in the solid components along with the liquid components (Fig 4) warrants further study since it may have important implications when analyzing the risk for the different blood components to efficiently transmit DENV, as well as for its detection during blood screening. It is also possible that a higher proportion of infectious cell culture supernatants would have been detected if subjected to additional passages in culture.

This situation does not have the same repercussions for DENV viral load determination in CCWB, since for that purpose, aliquots of CCWB were frozen after addition of Trizol as soon as the samples were received. Overall, the rates of RNA-reactivity for plasma specimens from the TMA-reactive subjects in the study were noticeably lower than those observed for the other components tested. While the original TMA was performed on plasma obtained from the EDTA whole blood testing tube, insufficient plasma remained following blood screening for us to extract and test by TaqMan. Rather, we had to use fresh frozen plasma from the blood unit. While precautions were taken to avoid formation of cryoprecipitate during thawing, it is possible that the lower proportion of reactives in the plasmas is due to poor recovery of virus associated with cryoprecipitate in some specimens. However, viral load determination for samples with Ct≤38 appears to have been unaffected or minimally affected by cryoprecipitate, since viral loads in reactive plasma specimens were comparable or in some cases higher than in the co-components. Therefore, comparison between DENV viral loads in plasma, serum and CCWB can be done since each specimen had been treated similarly, although different RNA extraction protocols were used for the liquid and cellular components analyzed and cryoprecipitate may have affected the rate of reactivity in plasma.

DENV viral load determinations in clots were performed after a variable time of storage (0.5–10 months) at 4°C and thus were subject to greater degradation over time. This may account for the lower average titers observed in this component when compared to other specimens (i.e., plasma, serum and CCWB), since this trend was not observed in the fresher clot samples available for analysis (S2 Fig). These findings are relevant for the blood transfusion setting, since packed RBCs for transfusion may be stored refrigerated at 4°C for up to 42 days after collection. Although the stability of virions in the clot may not reflect that in packed RBCs, these findings warrant further investigation of viral persistence and infectivity under long-term storage under proper conditions for transfusion.

Our results on DENV distribution in blood samples differed from the results reported when analyzing the distribution of the related viruses WNV, a flavivirus related to DENV, and HCV, another member of the family *Flaviviridae*. Our laboratory has previously demonstrated that WNV associates with RBCs in blood donors and WNV RNA can be detected in the cellular component of blood samples in concentrations up to 1-log_10_ higher than that observed in the plasma co-component of the same sample [28]. Conversely, for HCV, significantly more viral RNA was detected in plasma than in RBC specimens from the same collection [40]. The results on the distribution of DENV-1 and DENV-4 in blood samples from this study revealed that there are no significant differences in the detection of DENV viral RNA in individuals with quantifiable amounts of viral RNA in all tested components (S1 Table and Fig 2).

Although WNV association with human RBCs relative to plasma was significantly higher than the association of DENV or HCV with RBCs, it should be noted that in general, viral loads of WNV in human infections are much lower than those of DENV and HCV [41–44]. Taking into consideration the viral loads during the course of human infection for these viruses it is possible to speculate that the cellular compartment of the blood becomes saturated more quickly in human infection with DENV and HCV due to intense viral replication and release, resulting in higher viral loads in the plasma components relative to those seen in WNV infection, in which humans are an incidental host vs a required host for viral dissemination for DENV and HCV. Alternatively, ligands in the membrane of RBCs could have differing affinity to structural proteins of DENV (prM/M and E) than to structural proteins of WNV and HCV. Another explanation would be that HCV may also have higher affinity for proteins that are freely circulating in the plasma than either DENV or WNV, and binding competition would cause equilibrium in viral distribution.

Multiple studies have demonstrated an association of DENV viremia levels with disease severity, with patients with severe dengue generally having higher viral loads, but these studies of patients have not included asymptomatic infected individuals [44–47]. The infecting DENV virus type may also influence viremia levels in some populations [46, 48]. The samples used in this study represented only individual samples from a given infected individual, and as such, represent a snapshot of the infection and are not appropriate for determining peak viremia or viral persistence. However, the levels of DENV viremia observed in our specimens were within ranges previously reported for serum viremia in symptomatic individuals [44, 46].

Overall, the finding of comparable titers between different components from the same blood sample from DENV-1 and DENV-4 infected blood donors suggests that sensitivity of NAT for these viruses would not be improved by testing whole blood or components other than plasma. The detection of infectious DENV virions in all tested blood components including clots is congruent with previous findings of TT-DENV in all transfused components [13–16]. Additionally, the detection of DENV RNA and even infectious virions in clots after extended refrigerated storage suggests that this material has potential for use in testing when resources and sample sizes are limited.

## Supporting Information

S1 Table DENV RNA concentrations and infectivity for C6/36 cells in plasma, serum, whole blood and clot specimens from blood donors from Puerto Rico, 2012–2013, (n = 29). (DOC)

S2 Table DENV RNA concentrations and infectivity for C6/36 cells in plasma (PL), cellular component of whole blood (CCWB), and clot specimens from blood donors from Puerto Rico, 2012–2013, (n = 6). Selected samples were those with fresher clot specimens available.
(DOC)

S1 Fig Comparison of DENV-1 and DENV-4 viral loads in plasma and the cellular component of whole blood. A) DENV-1 (n = 19) and B) DENV-4 (n = 10) viral load averages observed in plasma (PL) and the cellular component of whole blood (CCWB) from samples from infected Puerto Rican blood donors, 2012–2013. Panels A and B illustrate side-by-side comparisons of average viral loads observed in each individual sample co-component (PL and CCWB). Whiskers represent 10-90th percentile. Outliers are shown as black dots. Mean is shown as a “+” sign and median as a horizontal line inside the box.
(TIF)

S2 Fig Comparisons of DENV-1 and DENV-4 viral loads in blood components. DENV-1 (n = 4) (panels A and B) and DENV-4 (n = 2) (panels C and D) viral load averages observed in plasma (PL), the cellular component of whole blood (CCWB) and clots (CL) from selected fresh samples from infected Puerto Rican blood donors, 2012–2013. Asterisk denotes that the plasma component of sample 57–13 did not yield DENV RNA. Panels A and C illustrate side-by-side comparisons of average viral loads observed in each individual sample co-component. Panels B and D shows the grouped results by component. Whiskers represent 10-90th percentile. Outliers are shown as black dots. Mean is shown as a “+” sign and median as a horizontal line inside the box.
(TIF)
