# Supplementary material for: Distribution of Dengue Virus Types 1 and 4 in Blood Components from Infected Blood Donors from Puerto Rico
Source: PLoS Negl Trop Dis. 2016 Feb 12;10(2):e0004445. doi: 10.1371/journal.pntd.0004445 (PMC4752498; doi:10.1371/journal.pntd.0004445)
Supplement: S1 Table — (DOC) [file pntd.0004445.s001.doc]

**Table S1. DENV RNA concentrations and infectivity for C6/36 cells in plasma, serum, whole blood and clot specimens from blood donors from Puerto Rico, 2012–2013, (n=29).**

| # | Sample | DENV RNA (Log_10_ PDU/ml ± SD) | | | DENV RNA  (Log_10_ PDU/mg ± SD), Clots | Age of clot (months) | Infectivity for C6/36 cells*,  FFA (Log_10_ FFU/ml) | |
| --- | --- | --- | --- | --- | --- | --- | --- | --- |
|  |  | Plasma | CCWB | Serum |  |  |  |  |
|  |  |  |  |  |  |  | Plasma | CCWB |
|  | DENV-1 |  | | | | | | |
| 1 | ARC-25-12 | 5.218 ± 0.246 | 4.654 ± 0.282 | n.a. | 3.304 ± 0.057 | 10.07 | 3.574 | 6.813 |
| 2 | ARC-28-12 | 4.817 ± 0.163 | 4.021 ± 0.269 | 4.259 ± 0.390 | undetected | - | n.d. | 6.439 |
| 3 | ARC-31-12 | 4.102 ± 0.166 | 3.920 ± 0.321 | 3.621 ± 0.205 | 3.579 ± 0.294 | 9.13 | n.d. | undetected |
| 4 | ARC-35-12 | 6.826 ± 0.147 | 6.004 ± 0.203 | 6.606 ± 0.191 | 4.468 ± 0.056 | 8.90 | 5.290 | 7.550 |
| 5 | ARC-40-12 | 6.640 ± 0.315 | 6.398 ± 0.166 | 6.603 ± 0.124 | 5.965 ± 0.067 | 8.67 | undetected | 3.778 |
| 6 | ARC-48-12 | 5.357 ± 0.260 | 4.737 ± 0.174 | 4.059 ± 0.114 | 3.371 ± 0.406 | 7.97 | n.d. | n.d. |
| 7 | ARC-52-12 | 6.590 ± 0.238 | 6.160 ± 0.227 | 6.526 ± 0.051 | 4.521 ± 0.237 | 7.87 | 5.591 | n.d. |
| 8 | ARC-58-12 | 4.888 ± 0.188 | 4.116 ± 0.132 | 4.724 ± 0.169 | undetected | - | n.d. | n.d. |
| 9 | ARC-59-12 | 3.852 ± 0.120 | 3.928 ± 0.441 | Reactive *Ct*>38 | undetected | - | n.d. | n.d. |
| 10 | ARC-64-12 | 5.405 ± 0.205 | 4.782 ± 0.190 | 5.097 ± 0.199 | Reactive *Ct*>38 | - | undetected | n.d. |
| 11 | ARC-68-12 | 7.267 ± 0.202 | 6.396 ± 0.486 | n.a. | 5.100 ± 0.175 | 6.80 | 3.740 | 4.857 |
| 12 | ARC-73-12 | 6.569 ± 0.414 | 5.643 ± 0.502 | n.a. | 4.902 ± 0.164 | 6.60 | 5.574 | 6.176 |
| 13 | ARC-92-12 | 5.247 ± 0.102 | 4.153 ± 0.422 | 4.837 ± 0.227 | Reactive *Ct*>38 | - | 3.161 | undetected |
| 14 | ARC-94-12 | 4.499 ± 0.489 | 3.601 ± 0.396 | undetected | undetected | - | 6.342 | n.d. |
| 15 | ARC-98-12 | 3.968 ± 0.336 | 3.759 ± 0.255 | 3.655 ± 0.263 | undetected | - | n.d. | 1.000 |
| 16 | ARC-02-13 | 6.697 ± 0.446 | 6.140 ± 0.839 | 6.762 ± 0.222 | 5.681 ± 0.169 | 5.40 | 6.648 | 6.505 |
| 17 | ARC-12-13 | 6.076 ± 0.501 | 5.476 ± 0.516 | 4.056 ± 0.287 | 5.151 ± 0.085 | 4.93 | 6.531 | 5.607 |
| 18 | ARC-16-13 | 5.601 ± 0.514 | 5.263 ± 0.198 | 5.241 ± 0.506 | 3.725 ± 0.055 | 4.93 | 3.672 | 2.243 |
| 19 | ARC-48-13 | 5.206 ± 0.153 | 4.074 ± 0.347 | 4.677 ± 0.040 | n.a. | - | undetected | n.d. |
|  | Average DENV-1 | 5.517 ± 1.010 | 5.052 ± 1.097 | 4.907 ± 0.959 | 4.524 ± 0.886 | 7.39 ± 1.70 | 5.01 ± 1.28 | 5.10 ± 2.02 |
|  | DENV-4 |  | | | | | | |
| 20 | ARC-55-12 | 4.652 ± 0.155 | 4.194 ± 0.295 | 4.042 ± 0.205 | 3.724 ± 0.277 | 7.63 | undetected | n.d. |
| 21 | ARC-69-12 | 4.988 ± 0.116 | 5.411 ± 0.313 | 5.185 ± 0.141 | 4.650 ± 0.069 | 6.80 | 4.462 | n.d. |
| 22 | ARC-75-12 | 7.999 ± 0.135 | 7.089 ± 0.171 | 7.423 ± 0.096 | 6.800 ± 0.008 | 6.60 | 3.512 | 5.597 |
| 23 | ARC-78-12 | 7.491 ± 0.122 | 6.788 ± 0.051 | undetected | 5.975 ± 0.072 | 6.73 | 2.301 | 4.550 |
| 24 | ARC-80-12 | 6.492 ± 0.113 | 5.264 ± 0.341 | n.a. | 4.516 ± 0.039 | 6.43 | 3.677 | 5.568 |
| 25 | ARC-89-12 | 6.248 ± 0.143 | 5.453 ± 0.080 | undetected | 4.067 ± 0.061 | 6.20 | 4.312 | 4.638 |
| 26 | ARC-01-13 | 5.396 ± 0.297 | 5.148 ± 0.302 | 5.791 ± 0.123 | 4.294 ± 0.115 | 5.53 | 3.699 | 4.161 |
| 27 | ARC-30-13 | 3.432 ± 0.360 | 4.040 ± 0.167 | 4.371 ± 0.135 | 4.266 ± 0.124 | 4.47 | 2.602 | n.d. |
| 28 | ARC-33-13 | 6.704 ± 0.114 | 6.501 ± 0.226 | 7.264 ± 0.123 | 6.201 ± 0.006 | 4.23 | undetected | 4.512 |
| 29 | ARC-43-13 | 3.522 ± 0.270 | 3.692 ± 0.204 | 3.607 ± 0.316 | 3.572 ± 0.176 | 3.53 | n.d. | n.d. |
|  | Average DENV-4 | 5.692 ± 1.484 | 5.383 ± 1.408 | 5.358 ± 1.108 | 4.806 ± 1.058 | 5.82 ± 1.26 | 3.51 ± 0.75 | 4.84 ± 0.55 |

n.a. sample not available for testing, n.d. not done

* in a single passage, results from cell culture supernatant (day 7 post-infection)
