# Supplementary material for: Distribution of Dengue Virus Types 1 and 4 in Blood Components from Infected Blood Donors from Puerto Rico
Source: PLoS Negl Trop Dis. 2016 Feb 12;10(2):e0004445. doi: 10.1371/journal.pntd.0004445 (PMC4752498; doi:10.1371/journal.pntd.0004445)
Supplement: S2 Table — Selected samples were those with fresher clot specimens available. (DOC) [file pntd.0004445.s002.doc]

**Table S2. DENV RNA concentrations and infectivity for C6/36 cells in plasma (PL), cellular component of whole blood (CCWB), and clot specimens from blood donors from Puerto Rico, 2012–2013, (n=6). Selected samples were those with fresher clot specimens available.**

| # | Sample | DENV RNA (Log_10_ PDU/ml ± SD) | | DENV RNA  (Log_10_ PDU/mg ± SD), Clots | Age of clot (months) | Infectivity for C6/36 cells,  FFA (Log_10_ FFU/ml) | |
| --- | --- | --- | --- | --- | --- | --- | --- |
|  |  | Plasma | CCWB |  |  |  |  |
|  |  |  |  |  |  | Plasma | CCWB |
|  | **DENV-1** |  | | | | | |
| 1 | ARC-57-13 | Undetected | 5.216 ± 0.020 | 5.043 ± 0.144 | 2.17 | undetected | undetected |
| 2 | ARC-60-13 | 3.682 ± 0.146 | 4.129 ± 0.201 | 4.381 ± 0.251 | 1.93 | undetected | undetected |
| 3 | ARC-68-13 | 6.028 ± 0.422 | 5.419 ± 0.039 | 5.399 ± 0.142 | 0.53 | undetected | 3.724 |
| 4 | ARC-69-13 | 5.963 ± 0.242 | 6.148 ± 0.027 | 5.671 ± 0.194 | 0.63 | 2.929 | 5.643 |
|  | Average DENV-1 | 5.365 ± 1.074 | 5.228 ± 0.730 | 4.913 ± 0.471 | 1.32 ± 0.74 | 2.929 | 4.68 ± 0.96 |
|  | **DENV-4** |  | | | | | |
| 5 | ARC-65-13 | 6.206 ± 0.036 | 6.042 ± 0.017 | 6.145 ± 0.023 | 1.07 | undetected | 3.204 |
| 6 | ARC-67-13 | 4.230 ± 0.041 | 4.555 ± 0.109 | 4.801 ± 0.092 | 0.47 | undetected | undetected |
|  | Average DENV-4 | 5.547 ± 0.932 | 5.299 ± 0.748 | 5.473 ± 0.676 | 0.77 ± 0.30 | - | 3.204 |

* in a single passage, results from cell culture supernatant (day 7 post-infection)
