# Supplementary figures and images for: Distribution of Dengue Virus Types 1 and 4 in Blood Components from Infected Blood Donors from Puerto Rico
Source: PLoS Negl Trop Dis. 2016 Feb 12;10(2):e0004445. doi: 10.1371/journal.pntd.0004445 (PMC4752498; doi:10.1371/journal.pntd.0004445)

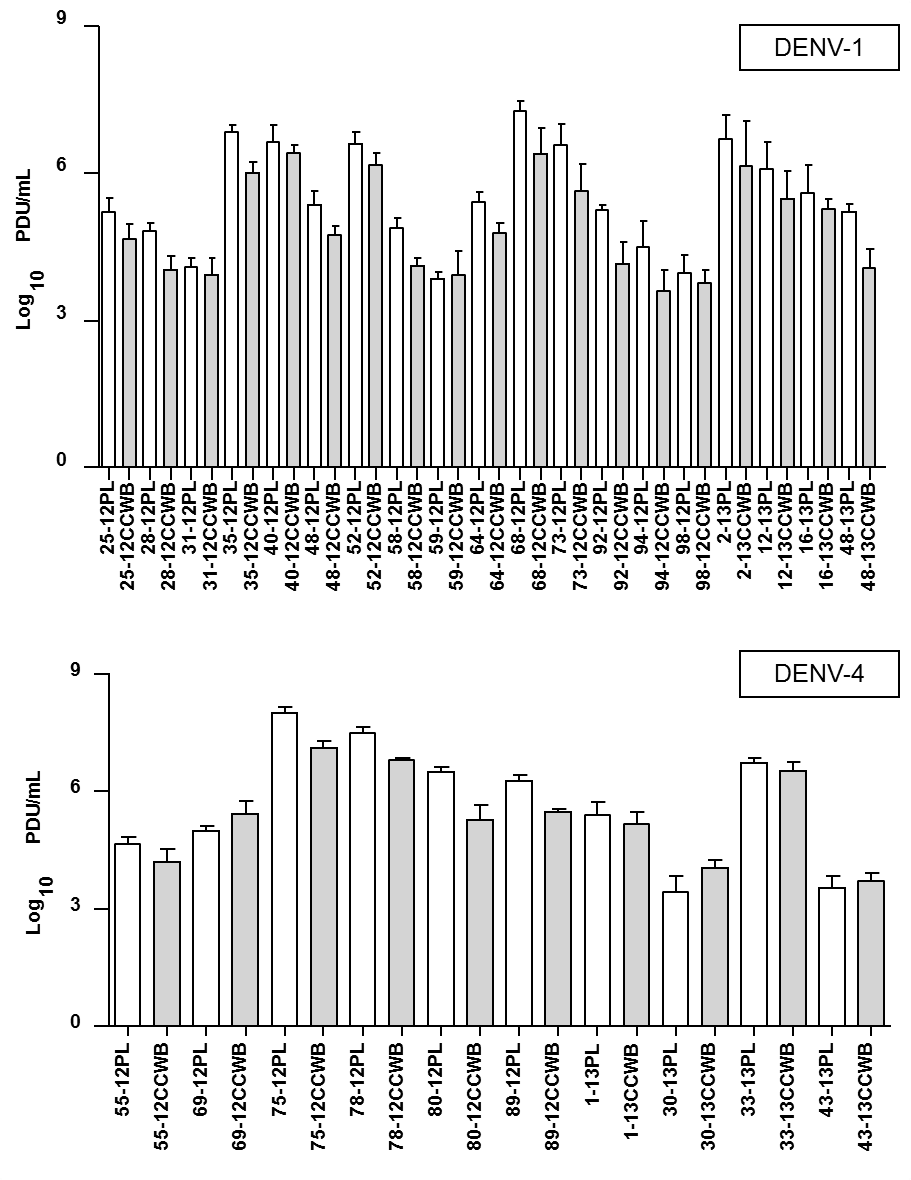

Supplement: S1 Fig — A) DENV-1 (n = 19) and B) DENV-4 (n = 10) viral load averages observed in plasma (PL) and the cellular component of whole blood (CCWB) from samples from infected Puerto Rican blood donors, 2012–2013. Panels A and B illustrate side-by-side comparisons of average viral loads observed in each individual sample co-component (PL and CCWB). Whiskers represent 10-90th percentile. Outliers are shown as black dots. Mean is shown as a “+” sign and median as a horizontal line inside the box. (TIF) [file pntd.0004445.s003.tif]

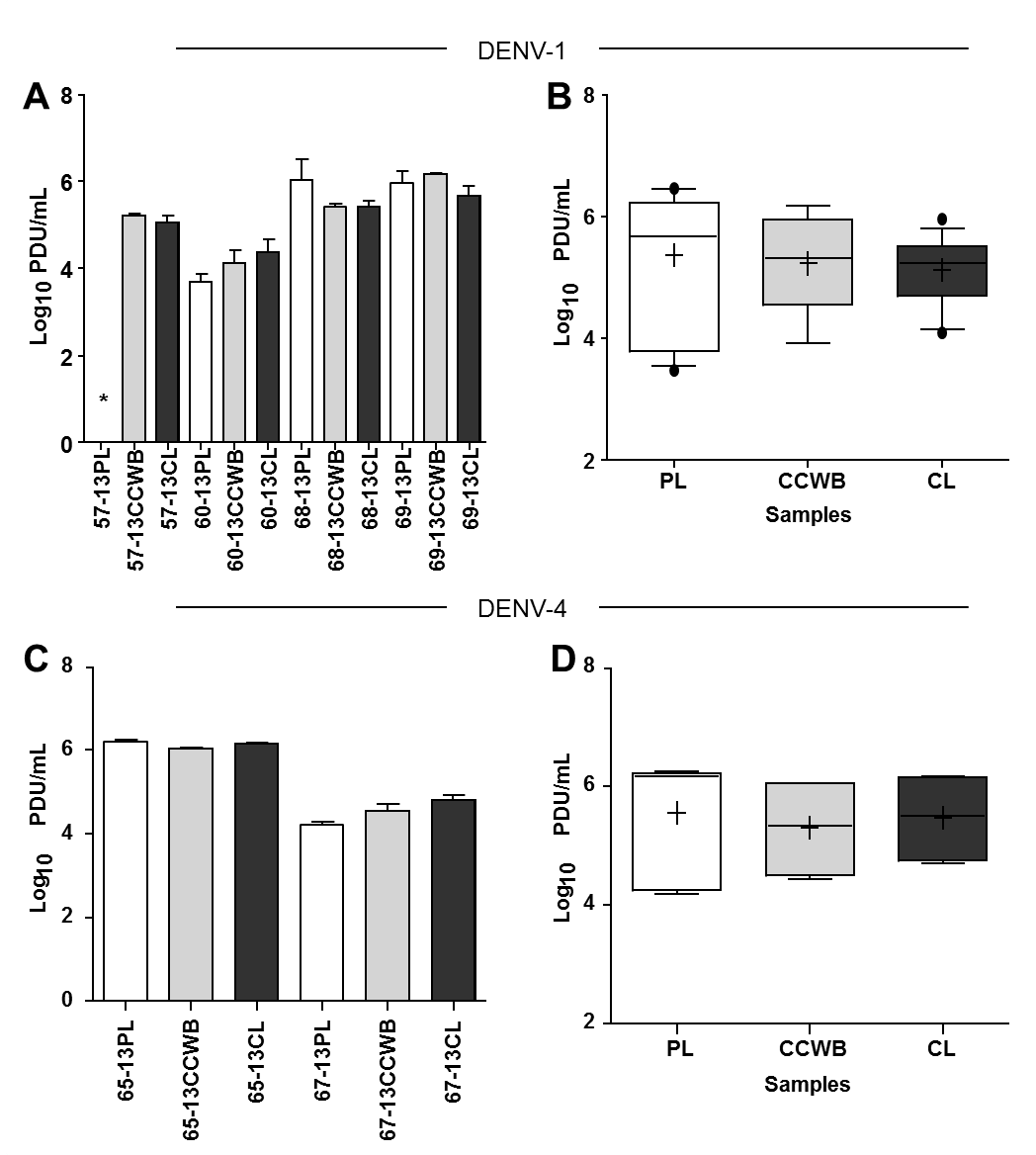

Supplement: S2 Fig — DENV-1 (n = 4) (panels A and B) and DENV-4 (n = 2) (panels C and D) viral load averages observed in plasma (PL), the cellular component of whole blood (CCWB) and clots (CL) from selected fresh samples from infected Puerto Rican blood donors, 2012–2013. Asterisk denotes that the plasma component of sample 57–13 did not yield DENV RNA. Panels A and C illustrate side-by-side comparisons of average viral loads observed in each individual sample co-component. Panels B and D shows the grouped results by component. Whiskers represent 10-90th percentile. Outliers are shown as black dots. Mean is shown as a “+” sign and median as a horizontal line inside the box. (TIF) [file pntd.0004445.s004.tif]
